# A *Young Seedling Stripe2* phenotype in rice is caused by mutation of a chloroplast-localized nucleoside diphosphate kinase 2 required for chloroplast biogenesis

**DOI:** 10.1590/1678-4685-GMB-2016-0267

**Published:** 2017-08-31

**Authors:** Kunneng Zhou, Jiafa Xia, Yuanlei Wang, Tingchen Ma, Zefu Li

**Affiliations:** 1 Key laboratory of Rice Genetics and Breeding, Rice Research Institute, Anhui Academy of Agricultural Sciences, Hefei, P.R. China

**Keywords:** Chloroplast biogenesis, NDPK, Oryza sativa, positional cloning, *YSS2* gene

## Abstract

Chloroplast development and chlorophyll (Chl) biosynthesis in plants are regulated by many genes, but the underlying molecular mechanisms remain largely elusive. We isolated a rice mutant named *yss2* (*young seedling stripe2*) with a striated seedling phenotype beginning from leaf 2 of delayed plant growth. The mutant developed normal green leaves from leaf 5, but reduced tillering and chlorotic leaves and panicles appeared later. Chlorotic *yss2* seedlings have decreased pigment contents and impaired chloroplast development. Genetic analysis showed that the mutant phenotype was due to a single recessive gene. Positional cloning and sequence analysis identified a single nucleotide substitution in *YSS2* gene causing an amino acid change from Gly to Asp. The *YSS2* allele encodes a NDPK2 (nucleoside diphosphate kinase 2) protein showing high similarity to other types of NDPKs. Real-time RT-PCR analysis demonstrated that *YSS2* transcripts accumulated highly in L4 sections at the early leaf development stage. Expression levels of genes associated with Chl biosynthesis and photosynthesis in *yss2* were mostly decreased, but genes involved in chloroplast biogenesis were up-regulated compared to the wild type. The YSS2 protein was associated with punctate structures in the chloroplasts of rice protoplasts. Our overall data suggest that YSS2 has important roles in chloroplast biogenesis.

## Introduction

Chloroplasts are essential organelles in higher plants. The formation of a mature chloroplast from a proplastid during plant development involves many steps: first, the development of the chloroplast itself followed by the development of a functional photosynthetic apparatus (Mullet, 1993; Sakamoto *et al.*, 2008). There are about 3,000 nuclear-encoded and nearly 120 plastid-encoded chloroplast proteins in higher plants (Timmis *et al.*, 2004; Reumann *et al.*, 2005; Pfalz and Pfannschmidt, 2013). These proteins play important roles in chloroplast development, photosynthesis and plastid transcription (Sakamoto *et al.*, 2008; Dong *et al.*, 2013; Pfalz and Pfannschmidt, 2013; Zhou *et al.*, 2013, 2017). Chloroplast biogenesis from proplastids to mature chloroplast goes through three steps (Kusumi *et al.*, 2010): first, plastid DNA synthesis and plastid division, second, establishment of the plastid transcription/translation apparatus, a key to chloroplast formation, and third, activation of the photosynthetic apparatus. At the molecular level, transcript accumulations from both nuclear and plastid genes are necessary for all three steps (Kusumi *et al.*, 2010). *FtsZ* (encoding a component of the plastid division machinery) is required for the first step (TerBush *et al.*, 2013); *RpoTp*, *rpoA* and *rpoB*, separately encoding NEP, and PEP α and β subunits, respectively, are highly expressed during the second step (Hricová *et al.*, 2006; Steiner *et al.*, 2011; Börner *et al.*, 2015), and *rbcS* (encoding the small subunit of ribulose-1,5-bisphosphate carboxylase), *rbcL* (encoding the large subunit of ribulose-1,5-bisphosphate carboxylase) and *psbA* (encoding the D1 subunit of the PSII complex) are abundant in the third step, which functions in activation of the photosynthetic apparatus (Hwang and Tabita, 1989; Nelson and Yocum, 2006). Chloroplast development is affected by alterations in expression levels of these genes. Similarly, *V1*, *V2*, *V3*, *St1* and *VYL* transcripts were reported to accumulate highly in the first or second steps of chloroplast differentiation and to be required for chloroplast biogenesis (Sugimoto *et al.*, 2004, 2007; Yoo *et al.*, 2009; Kusumi *et al.*, 2011; Dong *et al.*, 2013).

Chl-deficient mutants are ideal materials to study Chl biosynthesis, chloroplast development, and chloroplast RNA editing. *OsYGL1* encodes a Chl synthase that catalyzes esterification of chlorophyllide, the last step of Chl biosynthesis (Wu *et al.*, 2007). Knockdown of the *YGL2* gene, which encodes heme oxygenase 1, hinders Chl biosynthesis in rice (Chen *et al.*, 2013; Li *et al.*, 2013). A chloroplast PPR protein was encoded by the *WSL* gene, which regulated the splicing of *rpl2* transcript. In the *wsl* mutant, PEP-dependent plastid gene expression was obviously down-regulated, and plastid rRNAs synthesis and plastid translation efficiency were reduced. These led to the aberrant chloroplast development and sensitive response of the mutant to abiotic stress (Tan *et al.*, 2014). *YSS1* encodes a chloroplast nucleoid protein, which was characterized as an important regulator of PEP activity. Decreased accumulation of *YSS1* transcripts disrupted Chl biosynthesis and chloroplast differentiation (Zhou *et al.*, 2017). *OsWLP1* (encoding a chloroplast ribosome L13 protein) and *OsWP1* (encoding Val-tRNA synthetase) play essential roles in chloroplast development of leaf and panicle. Weak allele mutation in *WLP1* caused albino seedling phenotype and bleached panicles, especially severe under lower temperature. Chloroplast development could be affected in the seedlings and young panicles of *wlp1* mutant, although data are not available (Song *et al.*, 2013). Single nucleotide transitions in *OsWP1* lead to a severe albino phenotype (*wp1* died after the L4 stage) or a virescent phenotype (*wp1* showed striated leaf in seedlings and white panicles at heading). The *wp1* mutant arrested plastidic protein synthesis and biogenesis of chloroplast ribosomes and was defective in early chloroplast development (Wang *et al.*, 2016). In addition, *YSA*, *YLC1*, and *AM1* were also implicated in Chl biosynthesis and chloroplast formation in different ways (Su *et al.*, 2012; Zhou *et al.*, 2013; Sheng *et al.*, 2014).

NDPKs, which are among the oldest proteins and widely present in various prokaryotes and eukaryotes, mainly function in maintaining the metabolic balance between NTPs and NDPs in cells (Hasunuma *et al.*, 2003). They also have essential roles in cell growth and division, signal transduction and plant stress response (Zimmermann *et al.*, 1999; Moon *et al.*, 2003; Ryu *et al.*, 2005; Dorion *et al.*, 2006). Three kinds of NDPKs predominate in higher plants. NDPK1 is a cytoplasm-associated protein whereas NDPK2 and NDPK3 are separately localized in plastids and mitochondria (Bolter *et al.*, 2007; Kihara *et al.*, 2011). Different localizations suggest that NDPKs play important roles in different cell compartments. Previous studies demonstrated that NDPK2 is associated with embryo and seed development and involved in response to external stress (Yano *et al.*, 1995; Nato *et al.*, 1997; Kawasaki *et al.*, 2001). However, there is little reported evidence that NDPK2 participates in chloroplast development and Chl biosynthesis.

In this study, we aimed to characterize a young seedling stripe mutant, *yss2*. The mutant showed a striated phenotype from leaf 2 to leaf 4 and a normal leaf phenotype thereafter. Plant growth was also delayed and there were fewer tillers per plant than in the wild type. The chlorotic leaf phenotype is associated with decreased pigment levels and aberrant chloroplasts. At heading stage, the uppermost leaves are slightly chlorotic and immature panicles display a degree of whitening. We showed that the symptoms were caused by a mutation in a single gene locus that was fine-mapped to a 62.4 kb region in chromosome 12. Sequence analysis demonstrated that a single base mutation had occurred in the gene we named as *YSS2* and subsequently showed to encode a nucleoside diphosphate kinase 2 (NDPK2) with high similarity to NDPKs in other species. Expression analysis showed that *YSS2* was highly expressed in L4 tissues, the key time of chloroplast biogenesis. Subcellular localization showed that YSS2 is a chloroplast-associated protein. These results implied that *YSS2* plays important roles in chloroplast biogenesis.

## Materials and Methods

### Plant materials and growth conditions

The white leaf and panicle mutant *yss2* was identified in an MNU-mutagenized population of *japonica* cultivar Nongyuan 238. Plants were grown in a growth chamber or paddy fields. Crosses between the *yss2* mutant and Nongyuan 238 or Nanjing 11 were separately used for genetic analysis and gene mapping. Seeds of cultivars Nongyuan 238 and Nanjing 11 were obtained from the Chinese National Key Facility for Crop Gene Resources and Genetic Improvement in Beijing.

### Determination of pigment contents

Chls and Car were assayed spectrophotometrically according to methods described previously (Zhou *et al.*, 2013). Leaf samples were collected from second, third, fourth or fifth leaves at the L4 or L6 growth stages and separately marinated in 95% ethanol for 48 h in darkness. Absorbance of supernatants was measured with a DU 800 UV/Vis Spectrophotometer (Beckman Coulter) at 665, 649 and 470 nm.

### Transmission electron microscopy (TEM)

Leaf samples were prepared for TEM from green and white sections of third leaves in *yss2* mutant and from similar positions of wild type at the fully expanded L3 stage. Transverse sections of leaves were fixed in a solution of 2.5% glutaraldehyde and then incubated in 1% OsO_4_ overnight at 4 °C. After staining with uranyl acetate, tissues were dehydrated through an ethanol series, and embedded in Spurr's medium before ultrathin sectioning. Samples were air-dried, stained again and observed with a Hitachi H-7650 transmission electron microscope.

### Mapping of *YSS2*

Following initial mapping of the *YSS2* locus between Indel/SSR markers ID12-8 and RM3331 on chromosome 12 using 35 F_2_ mutant segregants, an additional 1,216 F_2_ plants with striated leaf phenotype were used for fine-mapping. High-density Indel markers were developed based on sequence differences between cv. Nipponbare (*japonica*) and 93-11 (*indica*). Primer pairs designed with Primer Premier 5.0 are listed in Table 1. Full-length cDNA and genomic DNA of the predicted ORFs in wild type and *yss2* mutant were amplified and sequenced.

**Table 1:** Primer sequences used in this study.

Mapping primers		
Name	Forward sequence (5′-3′)	Reverse sequence (5′-3′)
ID12-7	GTGGCTGTTTAGGAGCGTTT	CAACCAAACAGCAATGCAAC
ID12-8	CCTAGTTCAGCTCCTGCTTACC	GCAGAAGAGAAGTTGTGTGTCG
F41-5	TGCTCGAAATAATTGCTTGTTGG	CATGGGCGGAAGGTAGGGA
F41-18	AGAGCAGGTAACATAAGCAAAC	ATCCGAATACTTCCCTCACA
F41-22	GAAGATTTCGCTCGCCTGTT	TCCATGTCGAACCATTAGCA
F41-25	CGATCATGGTGGTTGGTCA	CTGGGTTCAAATCTCAAAATTAGT
F41-35	TCTCCTCCTAGCCCTGTC	GGTGCGGATTAACCTATT
F41-37	CCGTATGCAAGGTTGGATAG	TATGACTTTGACCCTTTGCC
F41-44	AGCCAACTCCCAAGAACAA	CCCACGCTGAGTAGATGGT
F41-48	CCAAAGGGTGCTTATTATTTAGTC	GCTGCCTGTCTGCTTCCAT
F41-55	AAATCGTGGGAGGGAATAAACA	GCGCCGCCATCATTGACC
Primers for quantitative RT-PCR and subcellular localization		
Name	Forward sequence (5′-3′)	Reverse sequence (5′-3′)
YSS2-RT1	GCGTCGTCGGTTGAGCAAT	AGTGCTCCTGTGCCAAGTCC
PORA	ATCACCAAGGGCTACGTCTC	GAGTTGTTGTTCCAGCTCCA
HEMA1	CACCAGTCTGAATCATAT	CTACCACTTCTCTAATCC
YGL1	TGGACAGTTGAAGATGTT	GAATAGGACGGTAAGGTT
CHLI	AGTAACCTTGGTGCTGTG	AATCCATCAACATTCAACTCTG
CHLH	CTATACATTCGCCACACT	TATCACACAACTCCCAAG
CHLD	GGAAAGAGAGGGCATTAG	CAATACGATCAAGTAAGTGTT
rbcL	GTTGAAAGGGATAAGTTGA	AATGGTTGTGAGTTTACG
rbcS	TCATCAGCTTCATCGCCTAC	ACTGGGAACACACGAAACAA
psbA	AAGTTTCTCTGATGGTATG	ATAGCACTGAATAGGGAA
LHCP	GTTCTCCATGTTCGGCTTCT	GACGAAGTTGGTGGCGTAG
psaB	TTGGTATTGCTACCGCACAT	CCGGACGTCCATAGAAAGAT
psbB	TCATATTGCTGCGGGTACAT	AGTTGCTGACCCATACCACA
psbC	TACAACCTTGGCAAGAACGA	TACGCCACCCACAGAATTTA
FtsZ	GTTGGTGTTTCTTCCAGCAA	CCTCAATAGACGACCCGATT
RpoTp	AAGTCTGGCTTACGCTGGTT	AGGATCCTCAGCATTCATCC
rpoA	AAATCGTTGATACGGCACAA	ATTCACATTTCGAACAGGCA
rpoB	GCATTGTTGGAACTGGATTG	GCCGATGGGTAACTAAAGGA
Ubq	GCTCCGTGGCGGTATCAT	CGGCAGTTGACAGCCCTAG
YSS2-GFP	TTTCTAGAATGGACGCCATGGCCGT	CGGGATCCCTCTACAAGCCATGGTGT

### Quantitative real-time RT-PCR

Total RNA was extracted from wild-type and *yss2* seedlings using an RNA Prep Pure Plant kit (Tiangen) and reverse-transcribed using a SuperScript II kit (TaKaRa). Real-time RT-PCR was performed using a SYBR^®^ Premix Ex Taq^TM^ kit (TaKaRa) on a LightCycler 480 Real-Time PCR System (Roche). The 2^-ΔΔCT^ method was used to analyze relative gene expression (Livak and Schmittgen, 2001). Primers for real-time RT-PCR (YSS2-RT1, PORA, HEMA1, YGL1, CHLI, CHLH, CHLD, rbcL, rbcS, psbA, LHCP, psaB, psbB, psbC, FtsZ, RpoTP, rpoA and rpoB), listed in Table 1, were designed by GenScript (https://www.genscript.com/ssl-bin/app/primer). The *ubiquitin* gene (*LOC_Os03g13170*) (Ubq) was used as a reference control.

### Sequence and phylogenetic analysis

Candidate genes were predicted by the RGAP database (http://rice.plantbiology.msu.edu/cgi-bin/gbrowse/rice/). Homologous sequences of YSS2 were identified using the blastp search mode at NCBI (http://www.ncbi.nlm.nih.gov/) and sequences were aligned with BioEdit software. A neighbor-joining tree based on 1,000 bootstrap replicates was generated using MEGA v4.1 software. Expression profiles of *YSS2*, *OsNDPK1* (*LOC_Os07g30970*) and *OsNDPK3* (*LOC_Os05g51700*) were obtained from the RiceXPro database (http://ricexpro.dna.affrc.go.jp/). Subcellular localization of YSS2 was predicted using the ChloroP (Emanuelsson *et al.*, 1999) and TargetP (Emanuelsson *et al.*, 2000) programs.

### Subcellular localization

The coding sequence of *YSS2* was amplified and cloned to the N-terminus of GFP in the transient expression vector pA7-GFP (primer pairs shown in Table 1). Fusion plasmid YSS2-GFP and free GFP were separately transformed into rice protoplasts and incubated in darkness at 28 °C for 16 h before examination (Chiu *et al.*, 1996; Chen *et al.*, 2006; Zhou *et al.*, 2013, 2017). GFP fluorescence was observed with a confocal laser scanning microscope (Carl Zeiss LSM700).

## Results

### Phenotypic characterization of the *yss2* mutant

**Figure 1:**
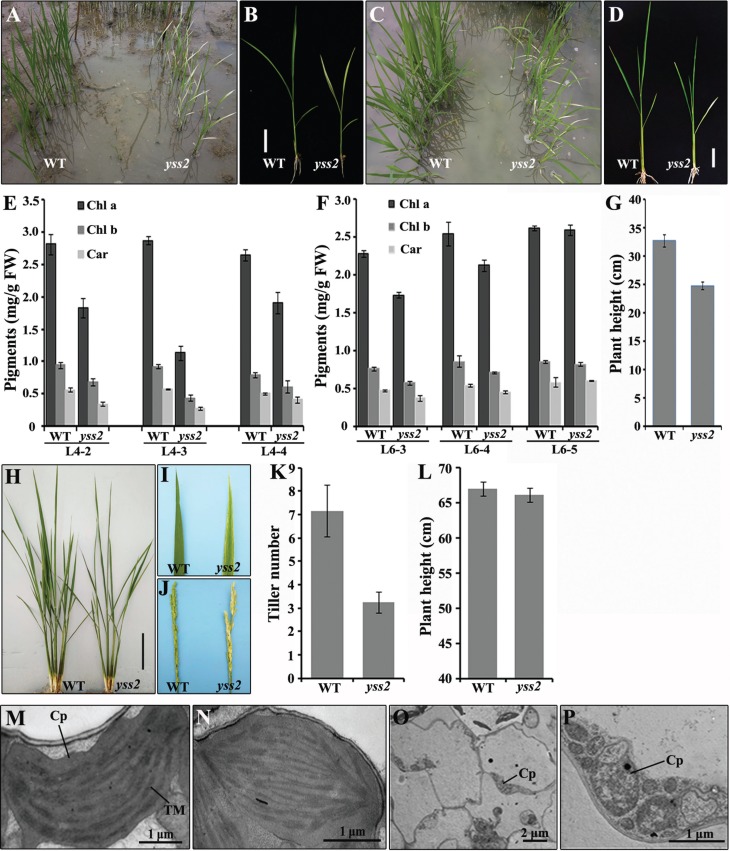
Phenotypic characteristics of the *yss2* mutant. Wild type and *yss2* mutant plants at the four-leaf (A, B) and six-leaf (C, D) stages in a paddy field. *Bars*, 5 cm. Pigment contents of the wild-type and *yss2* mutant plants in different leaf sections at the four-leaf (E) and six-leaf (F) stages (*e.g.*, L4-2, L4-3 separately represent the second and third leaves at the L4 growth stage). Values are means ± SD from three independent determinations. (G) Heights of wild type and *yss2* mutant at the six-leaf stage. Values are means ± SD from five independent repeats. (H) Phenotypes of wild type and *yss2* mutant at the maximum tillering stage. *Bar*, 10 cm. (I, J) The *yss2* mutant exhibiting striated leaves and white panicles after heading. (K, L) Tiller numbers and plant heights of wild type and *yss2* mutant after heading. Electron micrographs showing ultrastructures of chloroplasts from leaf 3 of wild type (M) as well as the green (N) and chlorotic sections (O, P) of leaf 3 in the *yss2* mutant at the three-leaf stage. Cp, chloroplast; TM, thylakoid membrane

The *yss2* mutant was originated from an *N*-methyl-*N*-nitrosourea (MNU) mutagenized population of *japonica* cultivar (cv.) Nongyuan 238. The mutant seedlings displayed a striated leaf phenotype in seedling leaves 2 to 4 under paddy field conditions (Figure 1A,B). However, the fifth and later leaves had normal green phenotype (Figure 1C,D). The *yss2* mutant showed delayed seedling growth compared to wild type (Figure 1A-D). To further characterize the mutant, we determined the pigment levels of chlorotic leaves at the four- and six-leaf stages. These leaves had reduced Chl a, Chl b and Car contents relative to the wild type (Figure 1E,F). However, from leaf 5 there was no obvious difference from wild type (Figure 1F). The height of *yss2* seedlings was less than the wild type (Figure 1A-D, G). At the maximum tillering stage, the *yss2* mutant was phenotypically similar to wild type except for reduced height (Figure 1H). The *yss2* mutant later developed a slight chlorotic leaf phenotype and white panicles at heading, but plant height was restored to the wild-type level (Figure 1I-L).

To investigate the effect of *yss2* on chloroplast development we compared the ultrastructures of chloroplasts in *yss2* mutant and wild type seedlings using TEM. The chloroplasts of green sections (basal section) of *yss2* leaves had well-developed lamellar structures with normally stacked grana and thylakoid membranes similar to wild type plants (Figure 1M,N); however, chloroplasts in the white segments were undifferentiated (Figure 1O,P). Collectively, our data showed that the *yss2* mutation caused a chlorotic defect that disrupted chloroplast development and delayed seedling growth.

### Cloning of the *YSS2* gene

For genetic analysis of the *YSS2* locus, reciprocal crosses between *yss2* mutant and Nongyuan 238 were made to determine the mode of inheritance of the *yss2* phenotype. F_1_ plants showed the wild type phenotype, and the F_2_ populations segregated 3 green : 1 stripe (Table 2). Thus the *yss2* phenotype was caused by a single recessive nuclear gene.

**Table 2:** Segregation of green and striated seedlings in F_2_ populations from two crosses.

Cross	No. green plants	No. stripe plants	χ^2^_3:1_ a
*yss2*/ Nongyuan238	455	140	0.686
Nongyuan238/ *yss2*	336	104	0.436
Pooled	791	244	0.446

a Value for significance at p = 0.05 and 1 *df* is 3.84

**Figure 2:**
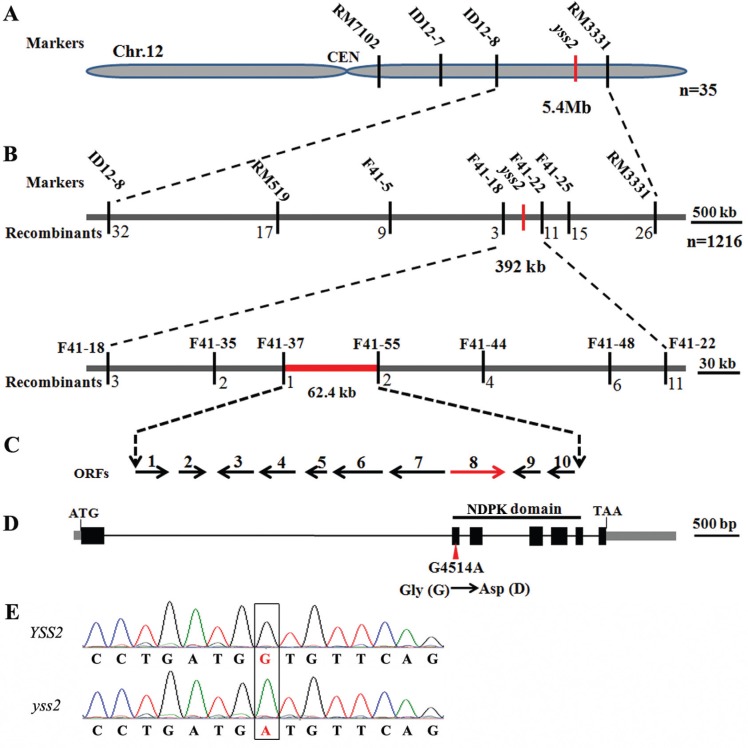
Positional cloning of the *YSS2* gene. (A) The *yss2* locus was mapped to a 5.4 Mb region between Indel/SSR markers ID12-8 and RM3331 on chromosome 12L. (B) The *yss2* locus was narrowed to a 62.4 kb interval between Indel markers F41-37 and F41-55 using 1,216 F_2_ homozygous mutant plants. (C) Ten ORFs were predicted in the region. (D) Schematic of ORF8 structure. ATG and TAA represent the start and stop codons, respectively. *Gray boxes* indicate 5' and 3' UTR. *Black boxes* and *lines* between them separately indicate exons and introns. The NDPK domain is indicated. The *yss2* mutant has a single G to D change in the eighth ORF. The mutation site is indicated by a red arrowhead. The single nucleotide change led to a Gly (G) to Asp (D) substitution. (E) Chromatograms from sequencing of wild type and *yss2* genomic DNA. *Black frame* indicates the mutation site

A mapping population was generated from cross *yss2*/ Nanjing 11. Thirty-five F_2_ individuals with typical *yss2* striated characteristics were used to map the *YSS2* locus to a 5.4 Mb region between markers ID12-8 and RM3331 on chromosome 12L (Figure 2A). With 1,216 homozygous F_2_ mutant individuals we narrowed the region to a 62.4 kb interval between Indel markers F41-37 and F41-55 (Figure 2B). The interval contained ten ORFs and the genes within it were predicted using the RGAP database (http://rice.plantbiology.msu.edu/) (Figure 2C, Table 3). Sequence analysis showed that the eighth ORF (designated nucleoside diphosphate kinase; LOC_Os12g36194) in the *yss2* mutant carried a single nucleotide change (G to A) in the second exon relative to wild type, resulting in an amino acid change from Gly to Asp at position 83 (Figure 2D,E). To verify the mutation in the *YSS2* gene, 15 green and five striated seedlings from the *yss2*/ Nanjing11 F_2_ population were sequenced and examined for presence of Gly or Asp at position 83. All five striated individuals carried only Asp, the four green seedlings carried only Gly, and the other 11 green seedlings were heterozygous, carrying both Gly and Asp. This provided evidence that the amino acid change was responsible for the mutant phenotype.

**Table 3:** Gene prediction within the 62.4 kb region delimited by markers.

Gene	Orientation	Annotation
LOC_Os12g36120	Forward	Retrotransposon protein, putative
LOC_Os12g36130	Forward	Expressed protein
LOC_Os12g36140	Reverse	Retrotransposon protein, putative
LOC_Os12g36150	Reverse	Retrotransposon protein, putative
LOC_Os12g36160	Reverse	Expressed protein
LOC_Os12g36170	Reverse	HEAT repeat family protein, putative
LOC_Os12g36180	Reverse	Auxilin, putative
**LOC_Os12g36194**	**Forward**	**Nucleoside diphosphate kinase, putative**
LOC_Os12g36210	Reverse	Inhibitor I family protein, putative
LOC_Os12g36220	Reverse	Inhibitor I family protein, putative

### *YSS2* encodes nucleoside diphosphate kinase 2

**Figure 3:**
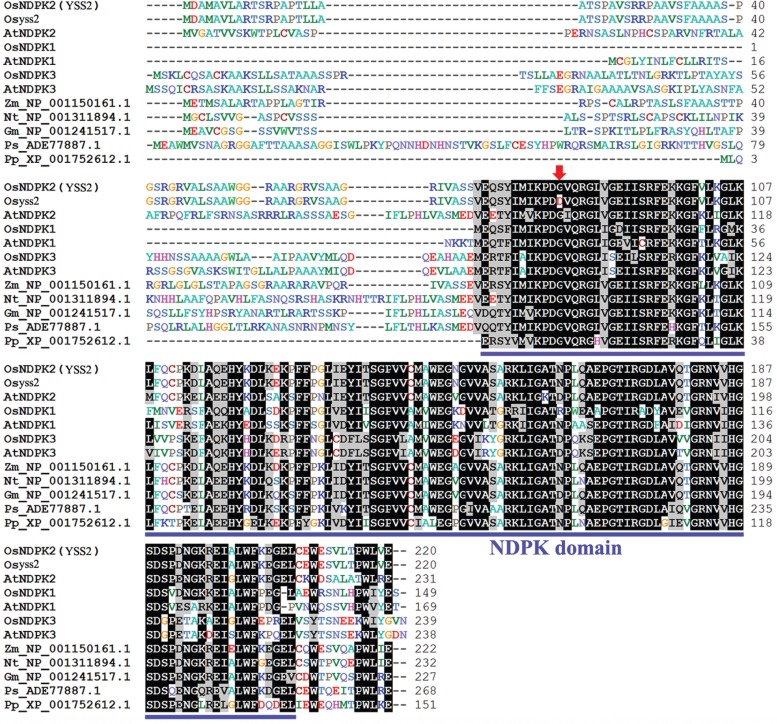
Amino acid sequence alignment of YSS2-related proteins. Conserved residues are shaded. Sequences are for OsNDPK2/YSS2 (*Oryza sativa*, LOC_Os12g36194), Osyss2 (the mutated YSS2 protein), AtNDPK2 (*Arabidopsis thaliana*, At5g63310), OsNDPK1 (*Oryza sativa*, LOC_Os07g30970), AtNDPK1 (*Arabidopsis thaliana*, NP_567346.1), OsNDPK3 (*Oryza sativa*, LOC_Os05g51700), AtNDPK3 (*Arabidopsis thaliana*, At4g11010), *Zea mays* NP_001150161.1, *Nicotiana tabacum* NP_001311894.1, *Glycine max* NP_001241517.1, *Picea sitchensis* ADE77887.1, *Physcomitrella patens* XP_001752612.1. Blue underlining represents the NDPK domain of NDPK-related proteins. A red arrow indicates the substituted amino acid in yss2.

The *YSS2* allele with seven exons and six introns encodes a polypeptide of 220 amino acid residues with a predicted molecular mass of 23.5 kDa (Figure 2D). The predicted structure indicated that the YSS2 protein contained an NDPK domain covering amino acid residues 73–207 (Figure 3). The YSS2 protein exhibited high similarity to NDPK superfamily proteins in other species across a region of nearly 150 amino acids in the C-terminus. Sequence alignment showed that the Gly^83^ site is highly conserved in NDPK proteins (Figure 3), suggesting that the site has an essential role.

**Figure 4:**
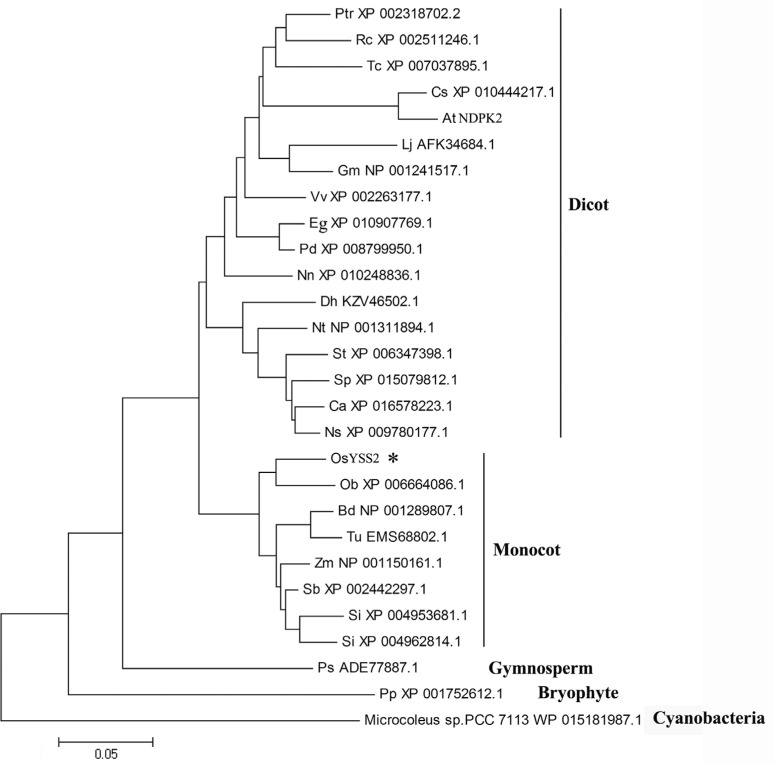
Phylogenetic analysis of YSS2 and its related proteins. OsYSS2 is indicated by an *asterisk*. Ptr, *Populus trichocarpa*; Rc, *Ricinus communis*; Tc, *Theobroma cacao*; Cs, *Camelina sativa*; At, *Arabidopsis thaliana*; Lj, *Lotus japonicas*; Gm, *Glycine max*; Vv, *Vitis vinifera*; Eg, *Elaeis guineensis*; Pd, *Phoenix dactylifera*; Nn, *Nelumbo nucifera*; Dh, *Dorcoceras hygrometricum*; Nt, *Nicotiana tabacum*; St, *Solanum tuberosum*; Sp, *Solanum pennellii*; Ca, *Capsicum annuum*; Ns, *Nicotiana sylvestris*; Os, *Oryza sativa*, Ob, *Oryza brachyantha*; Bd, *Brachypodium distachyon*; Tu, *Triticum urartu*; Zm, *Zea mays*; Sb, *Sorghum bicolor*; Si, *Setaria italic*; Ps, *Picea sitchensis*; Pp, *Physcomitrella patens*

Phylogenetic analysis revealed that YSS2-like proteins broadly exist in many photosynthetic organisms and likely evolved from the cyanobacteria to angiosperms, thereby forming a large subclade, in which orthologs from monocots and dicots are clearly separated (Figure 4). This suggests that YSS2 is an evolutionarily conserved protein evolved from prokaryotic to eukaryotic genomes.

### Gene expression analysis

**Figure 5:**
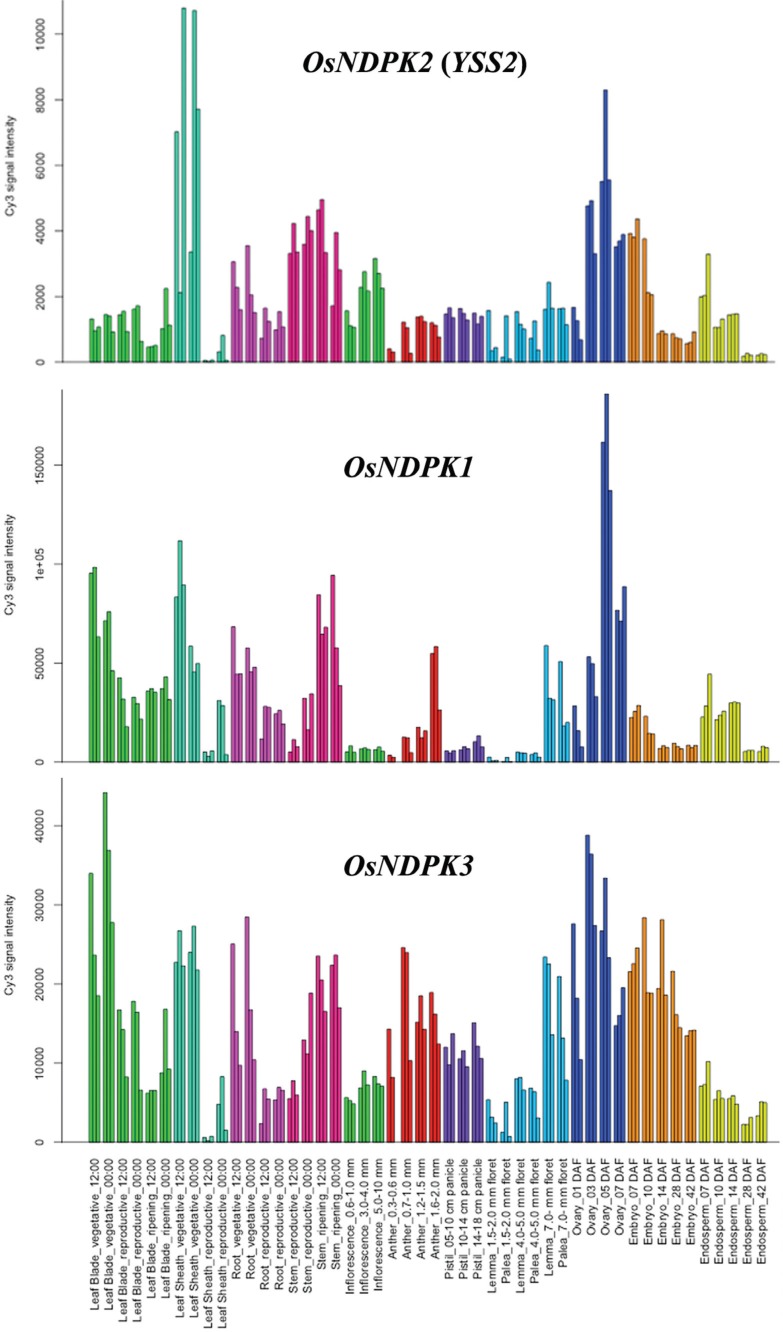
Expression analysis of *YSS2*, *OsNDPK1* and *OsNDPK3* at various growth stages. Data were collected from the rice expression profile database, RiceXPro.

The expression profiles of *OsYSS2*, *OsNDPK1* and *OsNDPK3* were predicted using the Rice Expression Profile Database. *YSS2* was expressed in various organs at different growth stages with higher levels in leaf sheaths, stems and ovaries. Overall expression levels were lower than for other NDPKs (Figure 5). *OsNDPK1* was mostly expressed in flag leaves, leaf sheaths and roots at the vegetative stage and in stems and ovaries at flowering. *OsNDPK3* was highly expressed in all tissues at different developmental stages (Figure 5).

**Figure 6:**
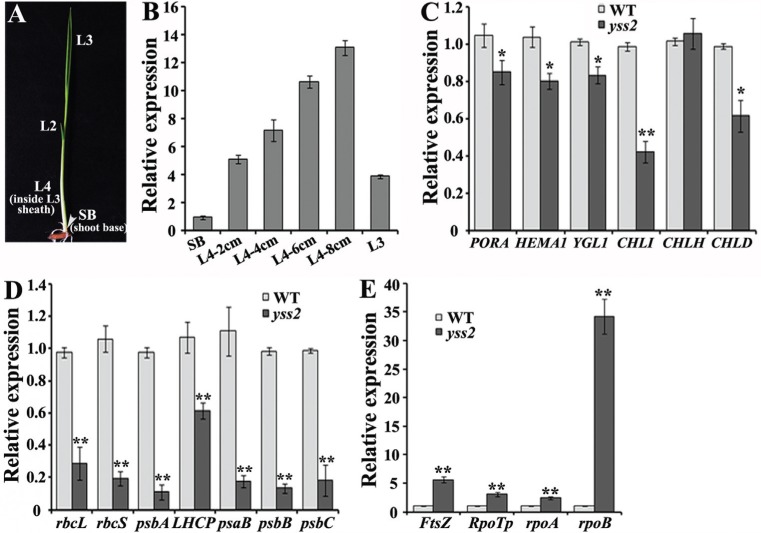
Expression analysis of *YSS2* and genes associated with Chl biosynthesis, photosynthesis and chloroplast biogenesis. (A) Diagram of rice seedling at the L3 stage in which the leaf 3 was fully expanded. SB (shoot base) indicates a 2 mm piece from the bottom of shoot. L2, L3 and L4 (inside L3 sheath) respectively indicate the second, third, and fourth leaf at the L3 stage. (B) Expression analysis of *YSS2* in SB, L4-2cm, L4-4cm, L4-6cm, L4-8cm, and L3 of wild type seedlings at the L3 stage grown in a paddy field. L4-2cm, L4-4cm, L4-6cm, and L4-8cm represent 2cm, 4cm, 6cm, and 8cm pieces from the bottom of shoot, respectively. (C, D and E) Real-time RT-PCR analysis of genes involved in Chl biosynthesis, photosynthesis and chloroplast biogenesis. Total RNA was extracted from leaf 3 of wild type and *yss2* mutant at the three-leaf stage in a paddy field. The *ubiquitin* gene was used as an internal control. Data are means ± SD of three replicates. **p = 0.01 and *p = 0.05, by Student's *t* test.

To investigate the expression profile of *YSS2* during the process of chloroplast biogenesis, we detected the *YSS2* transcripts in different sections of wild type seedlings at the L3 stage. The *YSS2* accumulated more in L4 tissues than in leaf 3 and shoot base, and the expression levels were gradually increased with the elongation of leaf 4 (Figure 6A,B). The data revealed that *YSS2* was highly expressed in the second step of chloroplast biogenesis.

Given the phenotypic difference between the *yss2* mutant and wild type seedlings, we compared the expression levels of genes associated with Chl biosynthesis, photosynthesis and chloroplast biogenesis. Expression levels of Chl biosynthesis-related genes, such as *PORA* (encoding NADPH-dependent protochlorophyllide oxidoreductase), *HEMA1* (encoding glutamyl tRNA reductase), *YGL1* (encoding Chl synthetase), *CHLI* and *CHLD* (encoding Mg-chelatase I and D subunits) were clearly decreased in *yss2* seedlings compared to wild type. However, there was no difference in expression of *CHLH* (encoding Mg-chelatase H subunit) (Figure 6C). Expression of genes involved in photosynthesis, such as *rbcL* and *rbcS* (encoding large and small subunits of Rubisco), *psbA*, *psbB* and *psbC* (encoding PSII subunits), *LHCP* (encoding PSII-associated light-harvesting chlorophyll protein) and *psaB* (encoding PSI subunit) was distinctly down-regulated in the mutant (Figure 6D). Genes required for the first (*FtsZ*, encoding a component of the plastid division machinery) and second (*RpoTP*, *rpoA* and *rpoB*, separately encoding NEP, and PEP α and β subunits) steps of chloroplast biosynthesis were up-regulated in the *yss2* mutant compared to wild type (Figure 6E). These data suggested that the *YSS2* is involved in the regulatory network of Chl biosynthesis and photosynthesis as well as chloroplast formation.

### Subcellular localization of YSS2

**Figure 7:**
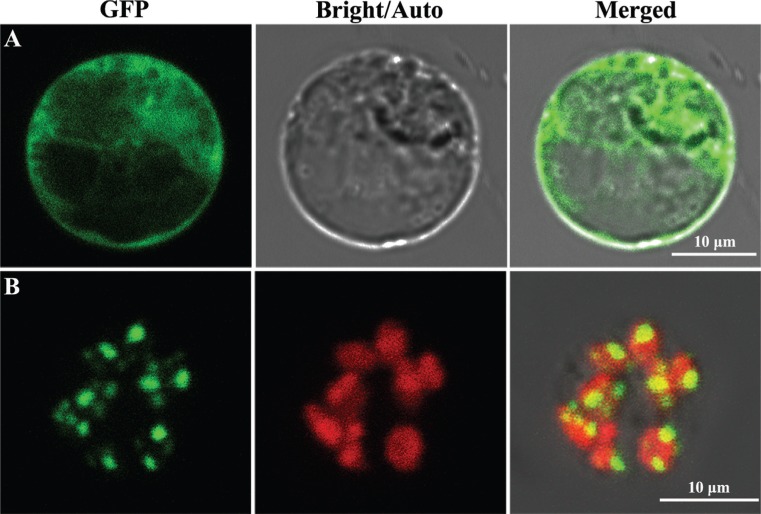
Subcellular location of YSS2. (A) Free GFP signals in rice protoplasts. (B) Transient expression of YSS2-GFP fusion proteins in rice protoplasts. GFP: GFP signals of free GFP and YSS2; Bright: bright field; Auto: chlorophyll autofluorescence; Merged: merged image.

TargetP (Emanuelsson *et al.*, 2000) and ChloroP (Emanuelsson *et al.*, 1999) softwares predicted that YSS2 localizes to chloroplasts and contains a chloroplast-targeting signal of 68 amino acid residues. To detect the actual localization of YSS2, free GFP and YSS2-GFP fusion proteins were each transiently expressed in rice protoplasts. Confocal microscopy confirmed that free GFP was dispersed in the cytoplasm (Figure 7A), whereas YSS2-GFP co-localized with Chl autofluorescence and displayed punctate structures (Figure 7B). The data showed that YSS2 is a chloroplast-associated protein.

## Discussion

A number of Chl-deficient and chloroplast development-associated mutants were recently identified in rice. Some of them show chlorotic phenotypes in young seedlings or young leaves and later develop normally, such as *ysa*, *ylc1*, *yss1* and *wsl* (Su *et al.*, 2012; Zhou *et al.*, 2013, 2017; Tan *et al.*, 2014). However, some mutants (*ygl1* and *vyl*) display the mutant phenotype and delayed plant growth throughout the entire life cycle (Wu *et al.*, 2007; Dong *et al.*, 2013). This contrasted with the *am1* mutant that also exhibited a chlorotic leaf phenotype throughout the life cycle but with little effect on plant development (Sheng *et al.*, 2014). The *ygl2* or *grc1* mutants showed yellow-green seedling leaf phenotypes but gradually reverted to almost normal green leaves from the tillering stage; both mutants showed delayed plant growth (Chen *et al.*, 2013; Li *et al.*, 2013). *wp1* and *wlp1* mutants both displayed chlorotic leaves accompanied by white panicles. The relatively weak *wp1* mutant had a virescent phenotype and developed white panicles (Wang *et al.*, 2016). The *wlp1* mutant produced albinic leaves until the four-leaf stage but became green at L4 and thereafter; a white panicle appeared at heading (Song *et al.*, 2013). The present white leaf and panicle mutant *yss2* showed a striated/chlorotic phenotype at L2, L3 and L4, but normal green leaves at leaf 5 and thereafter. The normal leaf phenotype in *yss2* persisted until maximum tillering, somewhat like *wlp1* mutant, a slight chlorotic leaf phenotype developed along with white panicles (Figure 1H-K). The chlorotic phenotype presented in young seedlings and panicles suggested that *YSS2* might play key roles in young tissues. Genes associated with plastid transcription/translation were largely regulated in *wlp1* mutant (Song *et al.*, 2013). Similarly, *wp1* impaired chloroplast ribosome biogenesis and reduced plastidic protein synthesis (Wang *et al.*, 2016). We observed that *yss2* mutant has phenotypes in seedlings and panicles similar to *wp1* and *wlp1*, suggesting that *YSS2* might function at the plastid transcription and translation stages, but further studies are needed for confirmation.

*YSS2* was mapped to a 62.4 kb interval on chromosome 12L and 10 ORFs were predicted in the region (Figure 2A-C). Genomic sequence analysis revealed that the only change in the mutant was in the 8^th^ ORF of *YSS2* and was a single base mutation causing an amino acid substitution of Gly by Asp at position 83 (Figure 2D,E). *YSS2* was highly expressed in the second step of chloroplast biogenesis, indicating that *YSS2* might directly participate in chloroplast formation. Subcellular localization showed that YSS2 is a chloroplast-associated protein (Figure 7B). These data implied that the *yss2* mutation might hinder chloroplast biogenesis during early leaf and panicle development, leading to the chlorotic phenotype in young seedlings and panicles. However, we cannot rule out the possibility that *YSS2* also has important roles in development of other types of plastids. This is supported by the fact that *YSS2* is expressed in non-green tissues (Figure 5). Although there was delayed plant growth and reduced height at the seedling stage the eventual plant height at maturity was similar to the wild type (Figure 1G,L), suggesting that *YSS2* paralogs sufficiently compensate for YSS2 function at some growth stages. Expression analysis showed that the housekeeping genes (*rpoA* and *rpoB*) and photosynthetic genes (such as *rbcL*, *psbA* and *psaB*) were separately up- and down-regulated in *yss2* mutant (Figure 6B,C), suggesting that *yss2* might decrease PEP activity and suppress plastid transcription. The dramatically elevated levels of *rpo* genes and decreased expression of photosynthetic genes indicated that the mutants lacked chloroplast ribosomes or reduced plastid DNA contents (Hess *et al.*, 1993; Udy *et al.*, 2012). We also observed that genes involved in Chl biosynthesis and chloroplast biogenesis were differently regulated (Figure 6C-E), implying that *YSS2* might have important roles in the regulatory network of both. Nevertheless, the reduced expression of genes for Chl biosynthesis is not strong and could be an indirect effect such as retrograde plastid-to-nucleus signaling that disturbs the expression of nuclear-encoded chloroplast genes (Mochizuki *et al.*, 2001; Nott *et al.*, 2006).

It has been reported that NDPKs regulate the metabolic balance between NTPs and NDPs by catalyzing the transfer of phosphate groups and are involved in cell growth and division, embryo and seed development, signal transduction and plant stress response (Yano *et al.*, 1995; Nato *et al.*, 1997; Zimmermann *et al.*, 1999; Moon *et al.*, 2003; Ryu *et al.*, 2005; Dorion *et al.*, 2006). Guanylate kinase (GK) functions in guanine nucleotide metabolism pathways by catalyzing the phosphorylation of (d)GMP to (d)GDP. GK is involved in maintenance of guanine nucleotide pools required for many metabolic processes. A rice GK gene, *V2*, was found to participate in chloroplast biogenesis (Sugimoto *et al.*, 2007). This provides the possibility that *YSS2* is involved in chloroplast biogenesis and that the *yss2* mutation disrupts the metabolic balance between NTPs and NDPs, a consequence of which is impaired photosynthesis and hindered plant growth. Sequence alignment and phylogenetic analysis revealed that YSS2 is an evolutionarily conserved protein with a NDPK domain. The amino acid Gly in the NDPK domain is highly conserved in all NDPK proteins (Figures 3 and 4). It seems that the amino acid change disrupts the integrity of the NDPK domain thereby affecting YSS2 function. OsNDPK1, OsNDPK2 and OsNDPK3 were reported to localize in cytoplasm, chloroplasts and mitochondria, respectively (Bolter *et al.*, 2007; Kihara *et al.*, 2011). These proteins share highly similar amino acid sequences in the NDPK domain, implying that NDPK proteins possess similar functions in different cell compartments. This is the first report that a NDPK protein participates in regulation of Chl biosynthesis and chloroplast biogenesis. Further research on the YSS2 protein could provide new insights into understanding how it participates in these functions, as well as in plant growth.
